# Glucocorticoid-induced osteoporosis is prevented by dietary prune in female mice

**DOI:** 10.3389/fcell.2023.1324649

**Published:** 2024-02-05

**Authors:** Nicholas J. Chargo, Kerri Neugebauer, Douglas V. Guzior, Robert A. Quinn, Narayanan Parameswaran, Laura R. McCabe

**Affiliations:** ^1^ Department of Physiology, Michigan State University, East Lansing, MI, United States; ^2^ College of Osteopathic Medicine, Michigan State University, East Lansing, MI, United States; ^3^ Department of Plant Soil and Microbiology, Michigan State University, East Lansing, MI, United States; ^4^ Department of Microbiology and Molecular Genetics, Michigan State University, East Lansing, MI, United States; ^5^ Department of Biochemistry and Molecular Biology, Michigan State University, East Lansing, MI, United States; ^6^ College of Human Medicine, Michigan State University, East Lansing, MI, United States

**Keywords:** prune, microbiota, bone, osteoporosis, gut, dysbiosis, bone volume, glucocorticoid

## Abstract

Glucocorticoid-induced osteoporosis (GIO) is a significant side effect of prolonged glucocorticoid (GC) treatment. Chronic GC treatment also leads to trabecular bone loss and gut microbiota dysbiosis in mice. The gut dysbiosis is mechanistically linked to GIO, which indicates that the microbiota can be targeted to prevent GIO. Prunes, a dried fruit and prebiotic, have emerged in the literature as an effective treatment for sex-steroid deficiency induced osteoporosis (primary osteoporosis). Prunes also significantly alter the composition of the gut microbiota in both rodent models and human studies. Therefore, we tested if dietary prune (DP) supplementation could prevent GC-induced bone loss and affect microbiota composition in an established model of GIO. Sixteen-week-old, skeletally mature, female C57BL/6J mice were treated with a subcutaneous 5 mg placebo or prednisolone pellet for 8 weeks and fed an AIN-93M control diet or a diet modified to include 5, 15, or 25% (w/w) dried California prune powder. As expected, GC treated mice developed significant trabecular bone loss in the distal femur. More importantly, as little as 5% DP supplementation effectively prevented trabecular bone loss. Further, dose dependent increases in trabecular bone volume fraction were observed in GC + 15% and GC + 25% DP mice. Amazingly, in the placebo (non-GC treated) groups, 25% DP supplementation caused a ∼3-fold increase in distal femur trabecular bone volume fraction; this sizable bone response has not been previously observed in healthy mice with gut targeted natural treatments. Along with the striking effect on bone health, GC treatment and 25% DP supplementation led to drastic shifts in gut microbiota composition and several specific changes are strongly associated with bone health. Taken together, these results are the first to demonstrate that DP supplementation effectively prevents the negative effects of prolonged GC therapy on trabecular bone health and strongly associates with shifts in the composition of the gut microbiota.

## Introduction

Glucocorticoids (GC) are potent and widely used anti-inflammatory drugs that treat several inflammatory conditions including rheumatoid arthritis, asthma, systemic lupus erythematosus, among many others (Overman et al., 2013). In the US, >1% of the population is currently undergoing chronic GC treatment to treat an inflammatory condition and provide symptomatic relief (Overman et al., 2013). Although GCs provide excellent clinical efficacy and successfully reduce inflammation, their long-term use can lead to several serious side effects including glucocorticoid-induced osteoporosis (GIO) (Adami and Saag, 2019; Gado et al., 2022). GIO is the most common cause of secondary osteoporosis and leads to an increased osteoporosis-related fracture risk and subsequent increases in morbidity and mortality (Amiche et al., 2016, 2018; Balasubramanian et al., 2018). Current strategies to combat GIO can be effective but have additional unwanted side effects leading to treatment discontinuation, poor adherence, and an increased fracture incidence (Compston, 2010; Lane, 2019; Chotiyarnwong and McCloskey, 2020; Iacopo et al., 2020; Laurent et al., 2022; Lim and Yeap, 2022). The best strategy to prevent GIO would be GC cessation; however, this is not an option for some patients. As such, there is a critical need to discover new strategies to prevent GIO and improve clinical outcomes (i.e., prevent fractures). In addition, natural, non-pharmacologic strategies are highly desirable as they carry very favorable side effect profiles, often lacking side effects all together.

The gut microbiota has been established as a critical target in the treatment of several diseases, including several etiologies of osteoporosis and importantly GIO (Sjögren et al., 2012; Villa et al., 2015; Chen et al., 2017; Quach and Britton, 2017; Rios-Arce et al., 2017; Pacifici, 2018; Hao et al., 2019; Schepper et al., 2020; Knudsen et al., 2021; Tu et al., 2021; Cronin et al., 2022; Chargo et al., 2023; Zhou et al., 2023). We have shown that chronic GC treatment alters the composition of the gut microbiota, leading to an imbalance, or dysbiosis, within the microbial community present (Schepper et al., 2020). Strategies to target and modulate the gut microbiota and potentially enhance bone health include ingesting probiotics (bacteria that confer a health benefit to the host) and/or prebiotics (substances that are metabolized by gut bacteria resulting in specific changes in composition and/or activity thus conferring health benefit to the host) (Hill et al., 2014; Davani-Davari et al., 2019). One example of a prebiotic that has recently garnered attention in the prevention of bone loss is dietary prune (Wallace, 2017).

Prunes, also known as dried plums, are fruits high in fiber, antioxidants, and bioactive compounds such as vitamins, amino acids, and phenolics (Wallace, 2017). Multiple studies demonstrate that dietary prune benefits bone health. Early studies comparing various dried fruit effects on bone health demonstrated that dietary prune (DP) supplementation was the most effective in preventing estrogen deficiency-induced bone loss in ovariectomized mice (Rendina et al., 2013). Additionally, DP supplementation can prevent, and even reverse, sex-steroid deficiency-induced bone loss (i.e., primary osteoporosis) (Deyhim et al., 2005; Franklin et al., 2006; Bu et al., 2007; Johnson et al., 2011; Rendina et al., 2012, 2013; Smith B. J. et al., 2014; Arjmandi et al., 2017; Smith et al., 2022) and alter gut microbiota composition in rodent models (Smith et al., 2022). Bone benefits have also been reported for healthy male C57BL/6J mice (Smith B. et al., 2014; Shahnazari et al., 2016) and for rodents receiving ionizing radiation or spinal cord injury (Schreurs et al., 2016; Liu et al., 2020).

Clinical benefits of DP translate to humans as well. Post-menopausal women that ingested 50–100 g of prunes per day for 6–12 months showed attenuated bone loss (Arjmandi et al., 2002, 2017; Hooshmand et al., 2011, 2016), with more modest effects in men (Hooshmand et al., 2011, 2014, 2016, 2021; Gaffen et al., 2019; Fajardo et al., 2020; Munoz et al., 2021). In addition, consumption of 50 g (∼6 prunes) per day for 1 year not only prevented further reductions in total hip bone mineral density (BMD) in post-menopausal women but was also associated with significant changes in gut microbiota composition (De Souza et al., 2022; Simpson et al., 2022). Taken together, there is significant evidence that DP supplementation causes shifts in gut microbiota composition and benefits bone health in both humans and animal models, especially in the context of primary osteoporosis.

Given its well-established benefits to bone and gastrointestinal health, we set out to examine if DP supplementation could prevent GIO, the leading cause of secondary osteoporosis. We tested the benefits of different DP concentrations (5, 15, 25% [w/w]) in an established subcutaneous 8-week-long GC treatment model. For the first time, we show that as little as 5% DP completely prevents GC-induced bone loss. Further, increases in DP concentration led to greater increases in bone volume and marked changes in microbiota composition that have not been previously reported. Interestingly, in healthy mice (control/placebo) we identified a ∼ 3-fold increase in distal femur trabecular bone volume fraction in mice with 25% DP supplementation, a response that correlates with microbiota changes. Overall, this study has important translational implications for the use of prunes as a therapeutic measure to maximize and maintain bone density during GC treatment.

## Materials and methods

### Diet preparation and nutrient composition

Prune (*Prunus domestica*) powder was provided by the California Prune Board (Roseville, CA, United States) directly from Sunsweet Growers, Inc (Yuba City, CA, United States). Briefly, prune powder was prepared by dehydrating and milling pitted prunes followed by screening for large particulate matter. Diets for all mouse experiments were purchased from Dyets, Inc (Bethlehem, PA, United States). Control groups were fed a standard AIN-93M rodent diet (DYET# 110900) and prune-treated groups were fed a modified AIN-93M diet containing 5, 15, or 25% (w/w, g/kg) prune powder. To remain isocaloric, prune powder was substituted for cornstarch in the control diet.

Nutritional composition was provided directly from the supplier (Table 1) and values are averages from multiple batches over a 5-year period. Samples of control and 25% prune diets along with multiple samples of prune powder were also sent to Creative Proteomics (Shirley, NY, United States) for flavonoid and phenolic acid quantification (Table 2). Briefly, 20 mg of each sample had flavonoids and phenolic acids extracted using 100% methanol and were measured and quantified against known standards using LC-MS/MS. Diet compositions were obtained directly from Dyets, Inc (Table 3). Proximate analyses were performed by Eurofins (Madison, WI, United States) on AIN-93M control and 25% prune diets (Table 3) to quantify total fat, carbohydrate, protein, and ash concentrations.

**TABLE 1:** Prune powder nutritional composition. Values for various nutritional components were provided by the supplier (Sunsweet Growers, Inc) and are presented as averages from several batches of prune powder over a 5-year period. Analyses were performed by an independent third-party testing facility. RAE = retinal activity equivalents.

	Per 100 g	Unit		Per 100 g	Unit
Moisture	3.65	**%**	**Vitamins and Minerals**		
Calories	383.8	**Kcal**	Vit A	941.50	**IU**
Total Fat	1.85	**g**	Vit A	40.30	**μg RAE**
Saturated Fat	1.59	**g**	Thiamine (B1)	0	**mg**
Monounsaturated Fat	0.05	**g**	Riboflavin (B2)	1.03	**mg**
Polyunsaturated Fat	0.13	**g**	Niacin (B3)	3.36	**mg**
Trans Fat	0	**g**	Pantothenic Acid (B5)	0.26	**mg**
Cholesterol	0	**g**	Vit B6	0.48	**mg**
Total Carbohydrates	88.02	**g**	Folic Acid	0	**μ** **g**
Total Dietary Fiber	16.74	**g**	Vit C	0	**mg**
Total Sugars	32.28	**g**	Vit D	0	**μ** **g**
Fructose	11.54	**g**	Vit E	2.01	**mg**
Glucose	19.16	**g**	Vit K	111.83	**μ** **g**
Sucrose	1.32	**g**	Boron	22.60	**ppm**
Sorbitol	18.16	**g**	Calcium	206.80	**mg**
Added Sugars	0	**g**	Copper	0	**mg**
Starch	0.28	**g**	Iron	0.92	**mg**
Protein	3.79	**g**	Magnesium	66.56	**mg**
Ash	2.69	**g**	Manganese	0	**mg**
**Typical Organic Acids**			Phosphorous	114.20	**mg**
Citric Acid	0.02	**g**	Potassium	1186.00	**mg**
Gluconic Acid	0	**g**	Sodium	0.79	**mg**
Malic Acid	0.45	**g**	Zinc	0	**mg**
Quinic Acid	3.73	**g**			

**TABLE 2:** Phenolic acid and flavonoid concentrations present in diets and prune powder. Prune powder and diet samples were sent to Creative Proteomics and phenolic acids and flavonoids were extracted using 100% methanol then analyzed using LC-MS/MS. Values are in mg/kg of sample. ND = not detected. Prune powder results are averages from three separate batches.

Compound (mg/kg)	AIN-93M control	Modified 25% prune	Prune powder
Apigenin	ND	ND	ND
Caffeic acid	1.228	1.283	2.028
Catechin	ND	ND	ND
Chlorogenic acid	0.016	16.713	66.880
Cinnamic acid	0.021	0.553	2.000
p-Coumaric acid	4.603	3.552	3.621
Daidzein	0.028	0.017	ND
Delphinidin	ND	ND	ND
Epicatechin	ND	ND	ND
Ferulic acid	1.739	1.352	1.007
Gallic acid	ND	0.098	0.264
Genistein	0.055	0.025	ND
Kaempferol	ND	ND	ND
Luteolin	0.001	0.009	0.029
Naringenin	0.007	0.024	0.048
Naringenin chalcone	ND	ND	ND
Proanthocyanidin A2	ND	ND	ND
Procyanidin B2	ND	ND	ND
Phloretin	ND	ND	ND
Protocatechuic acid	0.072	8.344	18.638
Quercetin-3-glucoside	0.003	0.145	0.557
Quercetin-3-galactoside	0.004	0.402	1.527
Quercetin	ND	0.050	0.182
Resveratrol	ND	ND	ND
Rutin	ND	4.204	18.661
Syringic acid	0.025	0.027	0.025
Vanillic acid	0.386	0.902	1.561

**TABLE 3:** Diet ingredient compositions and proximate analyses. Concentrations of ingredients present in AIN-93M Control and modified AIN-93M containing 5, 15, and 25% prune powder as provided by Dyets, Inc. Proximate analyses were performed by Eurofins for control and 25% prune diets to evaluate macronutrient content.

Ingredient (g/kg)	AIN-93M	Modified AIN-93M		
		5% prune	15% prune	25% prune
Casein	140	140	140	140
L-Cystine	1.8	1.8	1.8	1.8
Sucrose	100	100	100	100
Cornstarch	465.692	415.692	315.692	215.692
Prune Powder	0	50	150	250
Dyetrose	155	155	155	155
Soybean Oil	40	40	40	40
t-Butylhydroquinone	0.008	0.008	0.008	0.008
Cellulose	50	50	50	50
Mineral Mix (#210050)	35	35	35	35
Vitamin Mix (#310025)	10	10	10	10
Choline Bitartrate	2.5	2.5	2.5	2.5
**Proximate Analyses (g/100g)**				
Fat	4.4			4.2
Total Carbohydrates	67.4			67.1
Protein	12.3			11.4
Ash	2.46			2.77
Moisture **(%)**	13.4			14.5

### Animal studies

All animal procedures were approved by Michigan State University (MSU) Institutional Animal Care and Use Committee and complied with National Institutes of Health guidelines. Fifteen-week-old female C57BL/6J mice (n = 60, 10/group, Stock # 000664) were purchased from Jackson Laboratories (Bar Harbor, ME, United States). Upon arrival, mice were randomly divided and housed at 5 mice/cage, placed on sterilized standard chow (Teklad 2919, Inotiv, West Lafayette, IN, United States) and water *ad libitum*. At 16 weeks of age, after a 1-week acclimation period, cages were randomly selected and assigned to treatment groups. Mice were anesthetized via isoflurane inhalation and then had their dorsum shaved of hair and wiped with an alcohol prep wipe, and had either a 5 mg Prednisolone (GC, 60-day slow-release pellet, Innovative Research of America, Sarasota, FL, United States) or placebo pellet implanted subcutaneously (SQ) in the interscapular region using a trocar (Thiele et al., 2014; Schepper et al., 2020). This dosing equates to 3.9 mg/kg/day, on average. Mice recover from this procedure quickly and experience minimal pain/discomfort. One day following SQ pellet implantation, diets were switched to either AIN-93M (control) or a modified AIN-93M diet containing 5, 15, or 25% prune powder. Mice were maintained on their respective dietary treatment for 56 days (8-weeks) and were weighed, had cages changed, and food and water intake monitored weekly. After the 8-week treatment period, mice were humanely euthanized via inhaled isoflurane overdose with secondary confirmation via cervical dislocation and sterile blood, bones, colonic feces, and various tissues were collected for further analyses. Prior to euthanasia, a small amount of blood was collected from the tail vein for blood glucose measurement using a Metene blood glucose monitoring system (TD-4116, Metene Ltd., Walnut, CA, United States).

### Micro-computed tomography

Following euthanasia, femurs and vertebrae sections were collected and fixed in 10% neutral buffered formalin. After fixation for a minimum of 4 days, bones were stripped of soft tissue and transferred to 70% ethanol for scanning. All bones were scanned using a GE Explore Locus micro-computed tomography (μCT) system (GE Healthcare, Piscataway, NJ, United States) with a resolution of 20 μm obtained from 720 views. Each scan had femurs or vertebrae from both control and treatment groups and was phantom calibrated to standardize grayscale and maintain consistency. Prior to all femur analyses, the bone was aligned vertically, parallel to the *z*-axis. Analysis threshold was determined using auto thresholding features and isosurface visualization of trabecular bone sections. All bones were analyzed using a fixed threshold. Trabecular bone analyses were conducted in the femur in a region beginning immediately proximal to the distal metaphysis and extending 10% of the total bone length towards the diaphysis, excluding cortical bone. To assess bone microarchitecture at an axial site, we analyzed trabecular bone within the L4 vertebrae, again excluding cortical bone. Using GE Healthcare Microview software, we evaluated trabecular bone in the femur and vertebrae for the following measures: bone volume, total volume, trabecular spacing, trabecular number, trabecular thickness, bone mineral density, and bone mineral content. Trabecular bone volume is also reported as a function of body weight, as variations in body mass can influence trabecular bone density. Cortical measurements were obtained from the midshaft of the femur within a 2 × 2 × 2 mm region of interest that was centered at one-half of the total femur length (i.e., midshaft). Cortical measures included: mean thickness, periosteal perimeter, endocortical perimeter, total area, marrow area, cortical area, bone mineral density, and bone mineral content. All analyses were performed blinded to treatment group.

### 16S rRNA gene sequencing and microbiota analyses

Fecal pellets were collected from the colon during harvest, were snap frozen in liquid nitrogen, and stored at −80°C until further use. Fecal DNA was extracted from a single fecal pellet per mouse in ± GC and ± 25% DP diet groups using the DNeasy Powersoil Pro kit (Qiagen, Valencia, CA, United States) according to manufacturer protocol. Prior to submission for sequencing, DNA integrity was checked by amplifying the 16S rRNA gene via PCR with GoTaq Green Master Mix (Promega, Madison, WI, United States, M712B), and forward and reverse primers: 27F (5′-AGAGTTTGATCMTGGCTCAG-3′) and 1492R (5′-TACGGYTACCTTGTTACGACTT-3′). PCR amplification occurred under the following conditions: 5 min at 95°C; 30 cycles of 1 min at 95°C, 30 s at 48°C, 2 min at 72°C; 10 min at 72°C. The products were visualized via gel electrophoresis with 1% TAE agarose gel and compared to a 1 kb DNA ladder (Gold Biotechnology Inc., Olivette, MO, United States, D010-500).

DNA samples were submitted to the MSU RTSF Genomics Core for 16S rRNA gene-V4 hypervariable region amplicon library preparation and sequencing. The V4 hypervariable region of the 16S rRNA gene was amplified using primers 505F (5′-GTGCCAGCMGCCGCGGTAA-3′) and 806R (5′-GGACTACHVGGGTWTCTAAT-3′) following a previously developed protocol (Kozich et al., 2013). FastQ files were imported into Qiita (Gonzalez et al., 2018), a web-based GUI interface for QIIME2 (Bolyen et al., 2019). Following import, files were demultiplexed, trimmed at 200 bp, and filtered using Deblur (version 2021.9). Taxonomy was assigned using the q2-feature-classifier against the 99% SILVA 16S rRNA database (release 138) (Quast et al., 2013; Yilmaz et al., 2014). Alpha-diversity (i.e., Shannon Entropy) and beta-diversity (i.e., weighted-UniFrac distance) were calculated for the resulting table of amplified sequence variants (ASVs) in Qiita. Beta-diversity was visualized via principal coordinate analysis (PCoA) with difference between treatment groups tested via permutational analysis of variance (PERMANOVA) using the adonis function. PCoA plots were visualized by EMPeror (Vázquez-Baeza et al., 2013).

To determine which genera were most important in driving differences between groups, we used random forest machine learning. These analyses were performed in R (version 4.3.1) using the ‘randomForest’ package (version 4.7–1.1) (Liaw and Wiener, 2002). Top genera for treatment classification were determined by percent mean decrease in accuracy (MDA) while top general correlating with distal femur bone volume fraction (BV/TV%) were determined by increase in node purity (INP). Top genera for both analyses were further analyzed using Spearman correlations to determine their relation to bone health.

### Statistical analyses

Unless otherwise noted, all statistical analyses were completed using GraphPad Prism (version 9, GraphPad, San Diego, CA, United States). Comparisons between groups for all bone data and body parameters were completed via one-way ANOVA with post-hoc Tukey tests. If standard deviations were significantly different between groups, analyses were completed with Brown-Forsythe ANOVA with post-hoc Dunnett test. Food and water intake were analyzed via two-way ANOVA with post-hoc Tukey tests to determine significant differences between groups at each week. Differences in means of treatment groups for specific genera were analyzed via Kruskal–Wallis tests with post-hoc Dunn tests. Values in tables are presented as mean ± standard error of measurement (SEM). Box and whisker plots show the range of data with lines at the median and quartiles.

## Results

### Nutritional components and phenolic/flavonoid fingerprint of prune powder and diet compositions

The nutritional composition of prunes may vary depending upon the genotype, soil and climate. In our studies we used prunes (*Prunus domestica*) grown in California that were then dehydrated into a powder. For accuracy and reproducibility, we are including the nutritional components of the prune powder (Table 1), phenolic and flavonoid fingerprint of prune powder and diets (Table 2), and diet compositions (Table 3) to better describe the dietary prune (DP) intervention. The variation between batches was in the range of 10%–20% (data not shown).

### General body parameters are unaltered by GC treatment or dietary prune consumption

**FIGURE 1:**
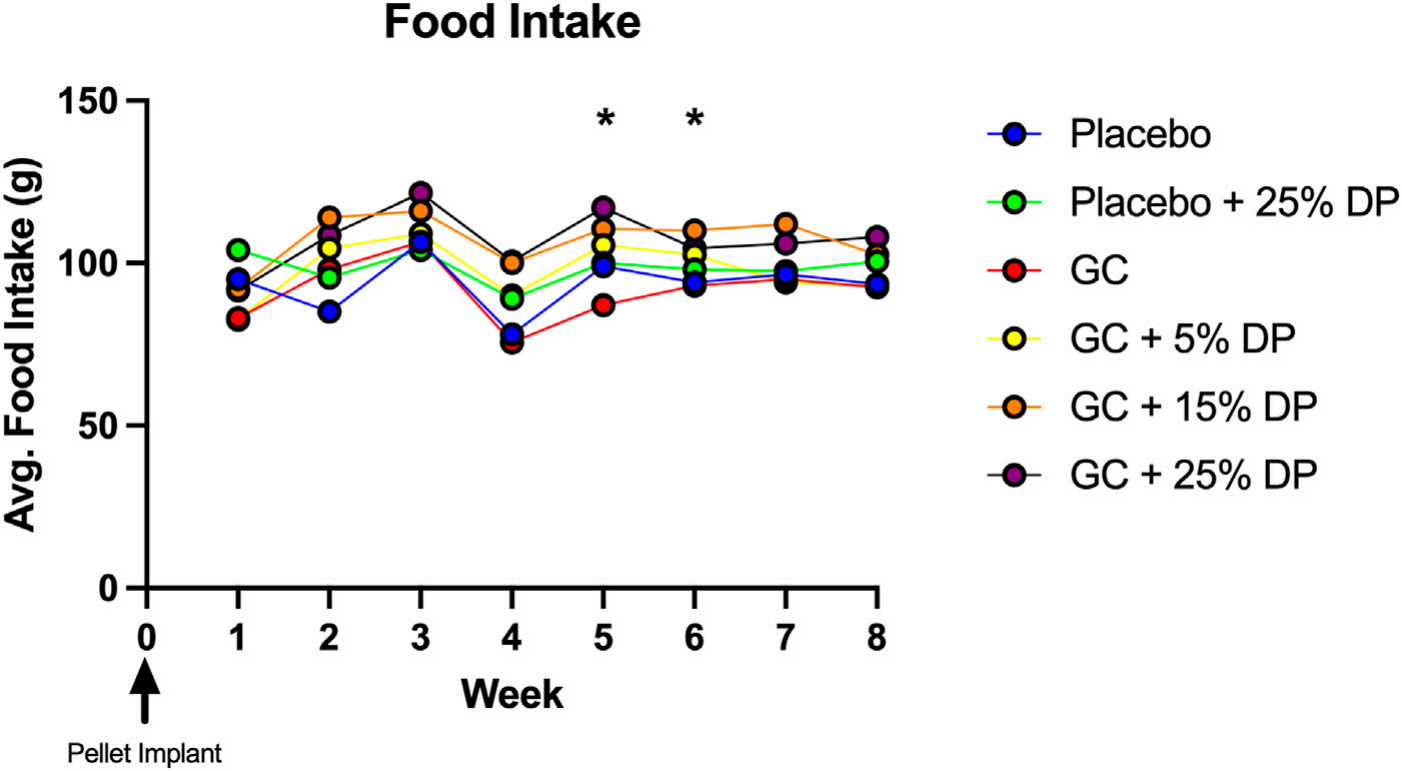
Weekly food intake. Food intake was measured weekly and demonstrates no pattern of difference between groups. GC + 25% DP (Week 5, *p* = 0.0087) and GC + 15% DP (Week 6, *p* = 0.0242) were both increased compared to GC. Statistical analyses performed via two-way ANOVA and post-hoc Tukey’s test to determine group differences within each week. n = 2 cages per group. DP = dietary prune, GC = glucocorticoid (Prednisolone).

Sixteen-week-old, skeletally mature, female C57BL/6J mice were treated with or without the glucocorticoid Prednisolone (GC) and with diets containing 0, 5, 15, or 25% prune powder (DP) for 8-weeks. At the end of the 8-week treatment period, there were no apparent effects on general body parameters or habitus (Table 4). This is evidenced by no significant differences in final body weight; liver, spleen, or kidney weight; or blood glucose levels. Of note, there were no consistent differences in food intake between any of the groups (Figure 1). However, during week 5, the GC + 25% DP group had increased food intake compared to GC alone (*p* = 0.0087) and during week 6, the GC + 15% DP group had increased food intake compared to GC alone (*p* = 0.0242). Since there are no patterns to these changes, it is likely that these findings are incidental.

**TABLE 4:** General body parameters. Values are presented as mean ± SEM. Statistical analyses completed via one-way ANOVA and post-hoc Tukey’s tests. Control indicates diets without prune supplementation. DP = dietary prune. n = 10 per group.

	Placebo		Glucocorticoid (prednisolone)			
Diet	Control	+ 25% DP	Control	+ 5% DP	+ 15% DP	+ 25% DP
Final Body Weight (g)	22.46 ± 0.72	22.98 ± 0.42	22.65 ± 0.54	22.33 ± 0.61	22.60 ± 0.68	23.27 ± 0.55
Liver Weight (g)	1.055 ± 0.09	0.9383 ± 0.07	0.9803 ± 0.05	0.9701 ± 0.05	0.969 ± 0.06	1.015 ± 0.06
Spleen Weight (g)	0.0804 ± 0.008	0.0806 ± 0.007	0.067 ± 0.006	0.0697 ± 0.012	0.0783 ± 0.007	0.0712 ± 0.009
Total Kidney Weight (g)	0.0891 ± 0.008	0.313 ± 0.006	0.2667 ± 0.01	0.2878 ± 0.021	0.2899 ± 0.011	0.3048 ± 0.011
Blood Glucose (mg/dL)	281.3 ± 33.67	275.0 ± 34.56	250.3 ± 25.55	259.7 ± 29.85	247.6 ± 14.47	251.2 ± 26.99

### Dietary prune supplement markedly increases trabecular bone volume and prevents distal femur glucocorticoid induced bone loss

**FIGURE 2:**
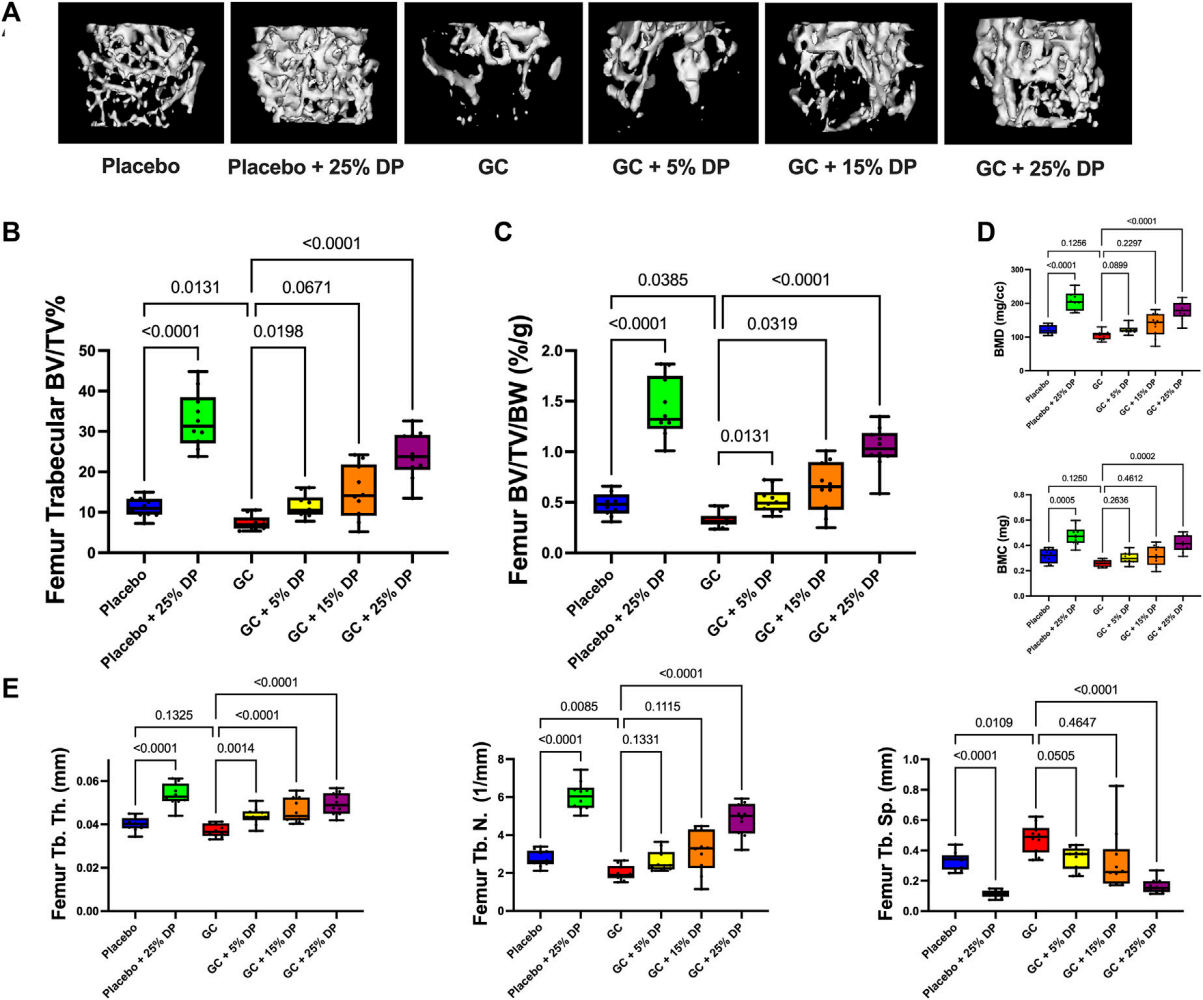
Dietary prune supplementation prevents distal femur trabecular GIO. 16-week-old female C57BL/6J mice were treated ± GC and ± prune supplemented diets (5, 15, and 25%) for 8 weeks. **(A)** Representative isosurface images of distal femur trabecular bone from each group. μCT analysis of distal femur trabecular bone expressed as **(B)** bone volume fraction and **(C)** corrected for body weight. **(D)** Distal femur trabecular bone mineral density and bone mineral content. **(E)** Distal femur trabecular bone microarchitectural analyses. n = 10/group. Box and whisker plots represent the range of the data with lines at the median and quartiles. Statistical analyses performed via One-Way ANOVA. GC = glucocorticoid (Prednisolone), DP = dietary prune, BV/TV% = bone volume/total volume, BV/TV/BW = bone volume/total volume/body weight, BMD = bone mineral density, BMC = bone mineral content, Tb. Th. = trabecular thickness, Tb. N. = trabecular number, Tb. Sp. = trabecular spacing.

Micro-computed tomography (μCT) analyses of distal femur trabecular bone revealed several significant changes. First, mice treated with GC while being fed a control diet displayed significant reductions in distal femur trabecular bone volume fraction (BV/TV%) compared to placebo treated mice on the control diet (*p* = 0.0131, Figure 2B). The same result was obtained after correcting for body weight (BV/TV/BW, *p* = 0.0385, Figure 2C). Impressively, supplementing the diet with as little as 5% DP significantly prevented distal femur trabecular GIO (*p* < 0.05, Figures 2B,C). BV/TV% was not significantly increased in the GC + 15% DP *versus* GC alone, likely due to variability, though there was strong trend (*p* = 0.0671). However, when corrected for body weight there was prevention of GIO (*p* = 0.0319, Figure 2C). With 25% DP GIO was significantly prevented (*p* < 0.0001, Figures 2B,C). It is important to note that 25% DP also increased BV/TV% and BV/TV/BW in the GC mice compared to Placebo controls (*p* < 0.001, Figures 2B,C). Even more interesting is that we observed a nearly 3-fold increase in BV/TV% and BV/TV/BW in the Placebo + 25% DP group compared to Placebo alone (*p* < 0.0001, Figures 2B,C). These results taken together indicate that supplementing the diet with prune in female mice effectively prevents distal femur GIO and leads to significant increases in distal femur bone volume fraction in healthy mice. The DP increases in BV/TV% were dose dependent, with increasing amounts of DP leading to higher trabecular bone volume fraction. Analysis of total femur length indicated that bone growth/lengths (which can impact trabecular measures) were similar between conditions, which further strengthens our results (Table 5). Translational measures of distal femur trabecular bone, bone mineral density (BMD) and bone mineral content (BMC), were also assessed. While there were not significant reductions in BMD and BMC resulting from GC treatment, prune supplementation, particularly 25% DP, increased both BMD and BMC (*p* < 0.001, Figure 2D).

**TABLE 5:** Femur mid-diaphyseal cortical parameters. μCT analyses of the femur mid-shaft after 8-weeks of treatment with ± GC and ± DP diets (5, 15, or 25%). Values are presented as mean ± SEM and analyses were completed via one-way ANOVA. Bolded GC value is significantly different from placebo (*p* = 0.0154) and bolded GC + 25% DP value is significantly different from GC (*p* = 0.0351). GC = glucocorticoid (Prednisolone), DP = dietary prune, Ct. Th. = cortical thickness, Ec. Pm. = endocortical perimeter, Ps. Pm. = periosteal perimeter, Ma. Ar. = marrow area, Ct. Ar. = cortical area, Tot. Ar. = total area, BMD = bone mineral density, BMC = bone mineral content.

	Placebo		Glucocorticoid (prednisolone)			
Diet	Control	+ 25% DP	Control	+ 5% DP	+ 15% DP	+ 25% DP
n	10	10	10	10	10	10
Femur Length (mm)	15.44 ± 0.12	15.08 ± 0.10	15.17 ± 0.06	15.38 ± 0.13	15.06 ± 0.10	15.13 ± 0.07
Ct. Th. (mm)	0.2411 ± 0.006	0.2432 ± 0.007	**0.2228 ± 0.005**	0.2306 ± 0.005	0.2276 ± 0.004	**0.2386 ± 0.004**
Ec. Pm. (mm)	3.482 ± 0.06	3.411 ± 0.02	3.583 ± 0.04	3.481 ± 0.03	3.423 ± 0.03	3.443 ± 0.03
Ps. Pm. (mm)	4.959 ± 0.06	4.921 ± 0.04	4.953 ± 0.03	4.905 ± 0.03	4.837 ± 0.04	4.925 ± 0.03
Ma. Ar. (mm^2^)	0.8567 ± 0.03	0.8151 ± 0.01	0.9006 ± 0.02	0.8578 ± 0.02	0.8309 ± 0.02	0.8392 ± 0.01
Ct. Ar. (mm^2^)	0.9630 ± 0.03	0.9517 ± 0.04	0.8919 ± 0.02	0.9146 ± 0.02	0.8923 ± 0.02	0.9481 ± 0.02
Tot. Ar. (mm^2^)	1.820 ± 0.04	1.767 ± 0.03	1.793 ± 0.02	1.772 ± 0.02	1.723 ± 0.03	1.787 ± 0.02
BMD (mg/cc)	856.1 ± 9.5	878.0 ± 15.0	890.0 ± 10.9	894.4 ± 11.0	885.2 ± 13.9	936.8 ± 8.24
BMC (mg)	0.01683 ± 0.0005	0.01626 ± 0.0004	0.01634 ± 0.0009	0.01638 ± 0.0005	0.01694 ± 0.0006	0.01684 ± 0.0005

Analyses of the distal femur trabecular bone microarchitecture indicates that GC treatment significantly reduced trabecular number (Tb. N.) and trended to reduce thickness (Tb. Th.) with significantly increased trabecular spacing (Tb. Sp., Figure 2E). GC mice treated with any concentration of DP exhibited increased Tb. Th. compared to GC alone. Only 25% DP prevented the GC reduction in Tb. N. although there was a trend with both the 5% DP and 15% DP treated groups. The GC-induced increase in Tb. Sp. was prevented by 5% DP and 25% DP while the 15% DP group was more variable and did not reach statistical significance. In the placebo group, 25% DP significantly increased Tb. N. and Tb. Th., and decreased Tb. Sp. compared to untreated placebo (Figure 2E). Taken together, 25% DP improves distal femur trabecular bone microarchitecture in female mice in both placebo and GC treated groups.

### Dietary prune effect on cortical bone parameters in the femur

To assess cortical bone, we analyzed the mid-shaft of the femur by μCT. After 8-weeks, there was a significant reduction in cortical thickness (Ct. Th.) in the GC group *versus* Placebo (*p* = 0.0154) and only the highest dose, 25%, DP prevented this reduction (*p* = 0.0351, Table 5). There were no differences in any other cortical measures including endocortical perimeter (Ec. Pm.), periosteal perimeter (Ps. Pm.), marrow area (Ma. Ar.), cortical area (Ct. Ar.), total area (Tot. Ar.), bone mineral density (BMD), and bone mineral content (BMC) (Table 5).

### Dietary prune improves vertebral trabecular bone microarchitecture

To assess an axial skeletal site, we analyzed trabecular bone within the L4 vertebrae by μCT. In the placebo + 25% DP group, trabecular bone parameters significantly improved as witnessed by increased BV/TV% (*p* < 0.0001, Supplementary Figure S1A), BV/TV/BW (*p* < 0.0001, Supplementary Figure S1B), BMD and BMC (*p* < 0.0001, *p* = 0.0027, Supplementary Figure S1C), and Tb. Th. (*p* < 0.0001, Supplementary Figure S1D) as well as decreased Tb. Sp. (*p* = 0.0028, Supplementary Figure S1D). There was no difference observed in Tb. N. These findings demonstrate that 25% DP supplementation in female mice improves trabecular bone microarchitecture in the vertebrae. We did not observe any bone loss in the vertebrae with GC treatment in this model but did see a beneficial dose-dependent bone response to DP in the GC treated groups. This included improvement in trabecular bone density and microarchitecture in the GC + 25% DP mice compared to GC alone for all measures except Tb. N. (Supplementary Figure S1). The GC + 15% DP mice benefited in most measures while GC + 5% DP mice showed improvement in BV/TV/BW but only trended to improve in all other measures (Supplementary Figure S1).

### Dietary prune significantly alters gut microbiome composition

**FIGURE 3:**
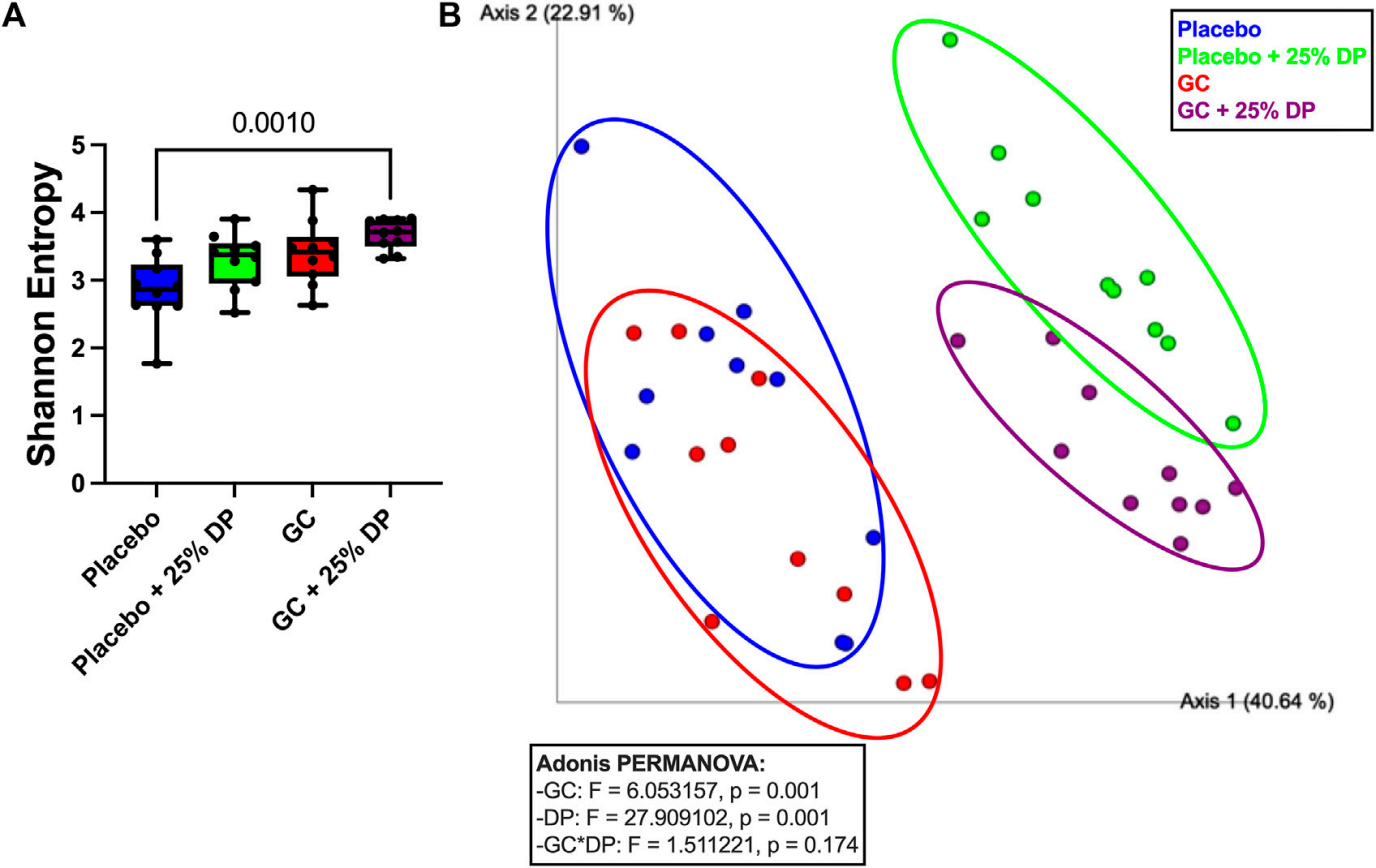
Glucocorticoid and dietary prune supplement alter gut microbiota composition. 16S rRNA gene sequencing was completed and broad level analyses were completed. **(A)** Alpha-diversity measured by the Shannon Entropy. Significance was first determined by Kruskal–Wallis test (*p* = 0.0025) with post-hoc Dunn’s tests. **(B)** Beta-diversity represented by principal coordinate analysis plot of weighted Unifrac distance matrices. Each point represents the compositional make up of one fecal sample (i.e., one mouse). Analyses completed via Adonis PERMANOVA. n = 10/group. GC = glucocorticoid, DP = dietary prune.

To determine the effect of DP supplementation on the composition of the gut microbiota, 16S rRNA gene sequencing was completed on the following four groups: placebo, placebo + 25% DP, GC, and GC + 25% DP. Differences in microbiome community structure were first examined via alpha- and beta-diversity. Our results show no significant differences in alpha-diversity, as measured via Shannon entropy, within placebo (±25% DP) or GC (±25% DP) groups. However, there is a significant increase in diversity within the community in the GC + 25% DP group compared to placebo (*p* = 0.0010, Figure 3A). To determine if there were significant differences in community between groups, weighted UniFrac distances were generated and visualized using principal coordinate analysis plots. PERMANOVA analysis identified significant differences between groups with regard to both DP (F = 27.909, *p* = 0.001) and GC (F = 6.053, *p* = 0.015) (Figure 3B). However, the interaction between DP and GC on community structure was not significant. Thus, differences in gut microbiota composition are caused by both GC and DP, although DP leads to more drastic shifts in composition than GC alone.

**FIGURE 4:**
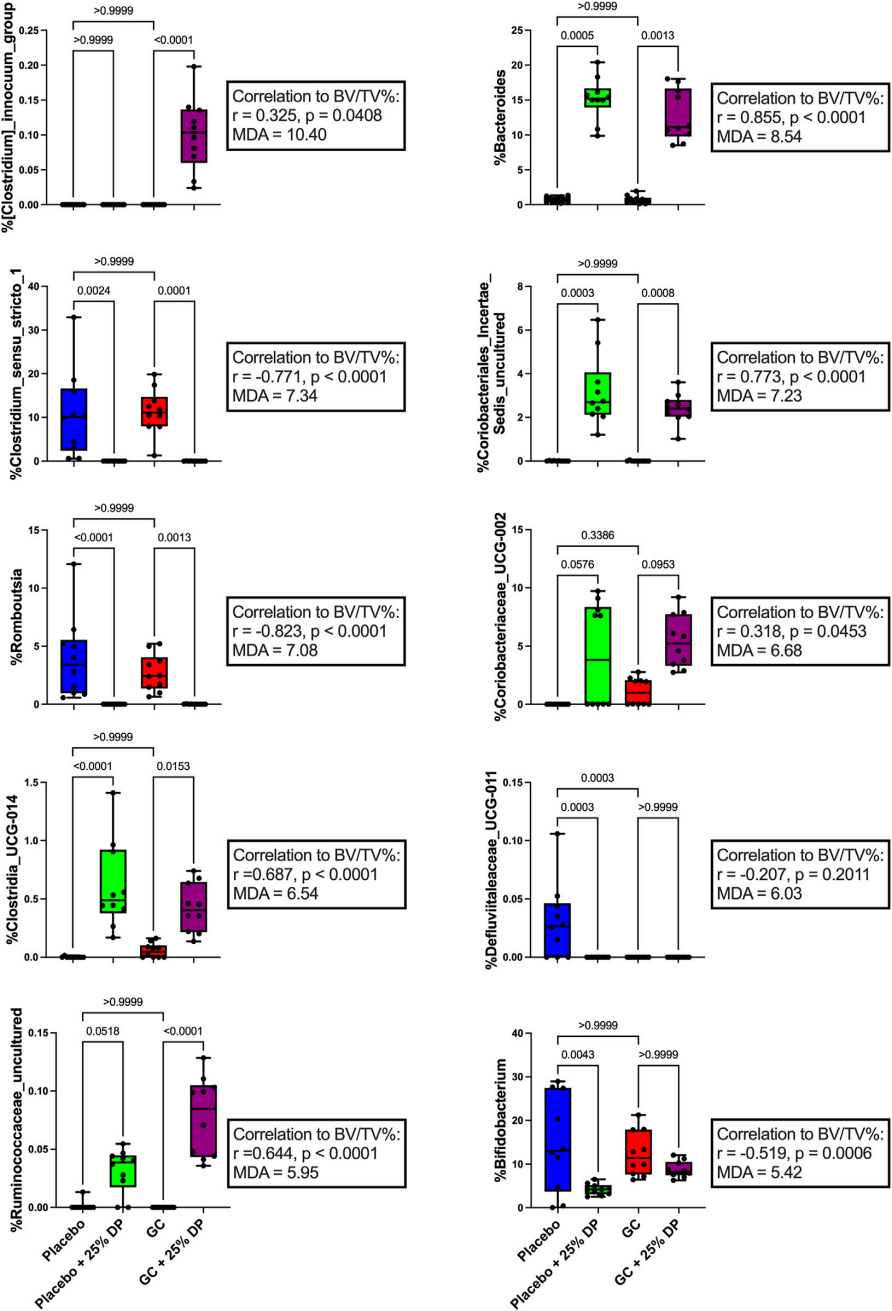
Dietary prune and GC treatment alter gut microbiota composition at the genus level. Selected bacterial taxa at the genus level from the random forest variable importance. Shown are the top 10 bacteria with the largest MDA values important in classifying samples into the appropriate groups. Individual taxa are analyzed via Kruskal–Wallis test and post-hoc Dunn’s tests. Spearman’s correlation analysis was performed to determine if there was a relationship between individual taxa and distal femur trabecular BV/TV%. n = 10 per group. DP = dietary prune, MDA = mean decrease accuracy, BV/TV% = bone volume/total volume.

Next, we examined specific gut microbiota changes by analyzing bacterial taxa at the genus level. Approximately 100 unique genera were identified by 16S rRNA gene sequencing, of which several were altered by DP and/or GC treatment. Random forest classification revealed several genera important in differentiating between groups. Overall classification error was 17.5%. However, classification error for placebo + 25% DP and GC + 25% DP groups was 0%, indicating that the microbiome of each group was distinctly unique. There was a 40% error rate classifying into the placebo alone and a 30% error rate classifying into GC alone, indicating that there is some overlap between the drug treatment groups on the control diet. The percent mean decrease accuracy (MDA) indicates how much the classification error rate increases when a specific feature is excluded from the random forest; ultimately, how much that feature contributes to overall model performance. The top ten genera that were most important in the overall classification model were *Clostridium innocuum group* (MDA = 10.40%)*, Bacteroides* (8.54)*, Clostridium sensu stricto 1* (7.34)*,* an unidentified genus in family *Coriobacteriales incertae sedis* (7.23)*, Romboutsia* (7.08)*, Coriobacteriaceae UCG-002* (6.68)*, Clostridia UCG-014* (6.54)*, Defluviitaleaceae UCG-011* (6.03), an unidentified genus in family *Ruminococcaceae* (5.95), and *Bifidobacterium* (5.42) There are several differences observed, with an overall theme of similar responses to increase or decrease a specific genus in response to GC or DP treatment. Spearman correlations uncovered several potential relationships between each genus and distal femur trabecular BV/TV% (Figure 4).

**FIGURE 5:**
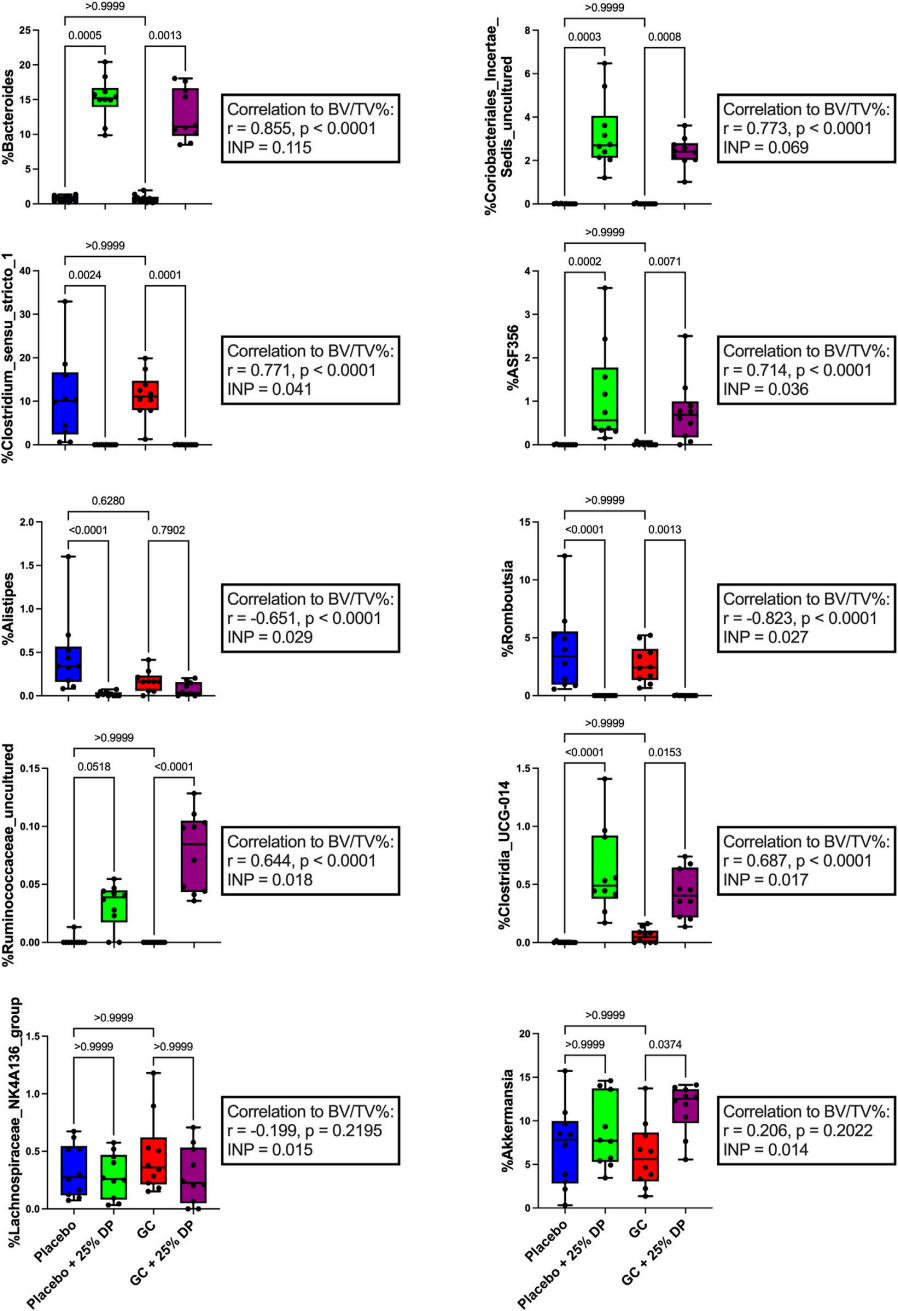
Changes in gut microbiota composition may help predict measures of bone health. Top ten individual bacterial genera ranked by increase in node purity (INC) from random forest regression by distal femur trabecular BV/TV%. Differences between groups in percent relative abundance in individual bacterial genera were analyzed via Kruskal–Wallis test and post-hoc Dunn’s tests. Spearman’s correlation analysis completed between each bacterial genera and distal femur trabecular BV/TV%. n = 10 per group. DP = dietary prune, BV/TV% = bone volume/total volume.

To further identify which bacterial taxa from the generated ASV data may predict distal femur trabecular BV/TV%, we used random forest regression. This resulted in 51.1% of variance in the microbiome being explained by BV/TV% with mean square error (MSE) = 0.006876627, root mean squared error (RMSE) = 0.08292543, and R^2^ = 0.5123688. Taxa were ranked using the increase in node purity (INP). The top ten bacterial genera from the random forest regression based on BV/TV% were *Bacteroides* (INP = 0.115), an unidentified genus in family *Coriobacteriales incertae sedis* (0.069), *Clostridium sensu stricto 1* (0.041), *ASF356* (0.036), *Alistipes* (0.029), *Romboutsia* (0.027), an unidentified genus in family *Ruminococcaceae* (0.018), *Clostridia UCG-014* (0.017), *Lachnospiraceae NK4A136 group* (0.015), and *Akkermansia* (0.014). Changes in percent relative abundance were graphed for each of these individual genera and had Spearman’s correlation analysis completed to identify the relationship with distal femur trabecular BV/TV% (Figure 5).

## Discussion

Glucocorticoid-induced osteoporosis is the number one cause of secondary osteoporosis and a significant side effect of chronic glucocorticoid therapy (Amiche et al., 2016). We demonstrate, for the first time, that DP supplementation as low as 5% effectively prevents GC-induced distal femoral trabecular bone loss in skeletally mature, female C57BL/6J mice. We have previously utilized gut targeted therapies to prevent several etiologies of bone loss in various models (Britton et al., 2014; Zhang et al., 2015; Collins et al., 2016; Raehtz et al., 2018; Schepper et al., 2019, 2020; Chargo et al., 2023; Kang et al., 2023), including two male mouse GIO models. In the subcutaneous GC treatment model, co-treatment with *Lactobacillus reuteri* ATCC 6475 (LR 6475) prevented GC-induced trabecular bone loss, gut barrier dysfunction, and shifted gut microbiota composition (Schepper et al., 2020). However, treating with LR 6475 only prevented bone loss from occurring and did not lead to bone improvements above that of the placebo treated controls. In the same study, we directly targeted the gut barrier by treating with a high molecular weight mucus supplement (MDY), which again led to prevention of GC-induced bone loss and gut barrier dysfunction, but not improvements beyond placebo treated controls. In an oral model of GIO (using growing male CD-1 mice), we also co-treated with LR 6475 and observed prevention of GC-induced bone loss and gut barrier dysfunction (Chargo et al., 2023). In an additional study using the oral GIO model in growing male CD-1 mice, we demonstrated that supplementing with an herbal supplement, Korean Red Ginseng extract, prevented GC-induced bone loss (Chargo et al., in review). Each of the above studies demonstrated prevention of bone loss by gut-targeted treatments. However, none of the treatments increased bone volume above the levels observed in the control mouse group. In comparison, DP supplementation in the GIO model not only prevented GC-induced bone loss but caused increases in bone volume up to 2-fold above that of placebo controls. This is a remarkably novel finding not previously observed with any of our gut targeted therapies in healthy animals or bone loss models. In addition, we observe similar beneficial effects of the prune diet in male mice, indicating that the effect is not sex dependent. Beneficial effects in male mice have been previously reported (Wallace, 2017) and we will expand upon this in future publications (in preparation).

Our results show that DP supplementation leads to drastic shifts in gut microbiota composition and that specific shifts in microbial community composition are strongly linked with trabecular bone health. Previous studies have investigated the effect of DP supplementation on gut microbiota composition in rodent models of primary osteoporosis and in post-menopausal women (Simpson et al., 2022; Smith et al., 2022). Prune supplementation led to drastic shifts in gut microbiota composition in both. In the rodent model, the authors observed significant shifts at the taxonomic level of family including increases in *Lachnospiraceae*, *S24-7*, *Coriobacteriaceae*, and decreases in *Bifidobacteriaceae* in the prune treated mice compared to controls (Smith et al., 2022). In our study, we observe the same response in families *Lachnospiraceae* and *Bifidobacteriaceae* (data not shown). We did not identify families *S24-7* or *Coriobacteriaceae*; these may have been present at very low abundances or may not have been present within our vivarium. In the human study, the women whose diets were supplemented with 50 g of prunes per day had increases in members of the *Lachnospiraceae* family, genus *Blautia*, and genus *Anaerostipes* and a reduction in order Oscillospirales (Simpson et al., 2022). We observe similar responses in family *Lachnospiraceae* but do not observe any differences in *Blautia* or Oscillospirales (data not shown). Overall, it appears that similar patterns of shifts in gut microbiota composition are observed not only between studies at different institutions in different models, but also translationally in humans. This indicates that results observed in pre-clinical rodent studies may be directly translatable to humans, further strengthening the rationale for using DP as an adjunct to GC therapy to prevent deleterious bone effects.

Results from the random forest analysis revealed several different bacterial taxa that were important in classifying samples into their respective groups (Figure 4) and predicting bone volume fraction (Figure 5). There is sparse literature regarding the relation of the top ten taxa from each analysis in the context of GIO as well as DP supplementation. Therefore, the results presented in this study are the first to describe each taxon not only in the context of GIO but in the context of GIO together with DP supplementation. Results revealed that the taxa were either increased or decreased in response to prune treatment and there were no significant differences between placebo and GC treated mice in the control or DP groups.

One of the top bacterial genera that changed in response to DP treatment in both the placebo and GC treated groups was *Bacteroides*. We observed a near 15% increase in both groups compared to their respective controls (Figures 4, 5) and there was a very strong significant positive correlation to distal femur trabecular BV/TV%. These findings indicate that there may be a relationship between increased *Bacteroides* abundance and improved bone health. In the random forest models, *Bacteroides* was the second most important genera (behind *Clostridium innocuum group*) in classifying samples into appropriate groups and the most important genera in the regression model predicting BV/TV%. *Bacteroides* is a genus of bacteria that has been studied extensively in humans, has associations with both health and several disease states, and includes species that are beneficial as well as pathogenic (Wexler, 2007; Zafar and Saier, 2021). Due to the high carbohydrate content (fiber, sugars, etc.) of the prune powder in the 25% prune diet, it is not surprising that we observe large increases in groups of bacteria (especially within *Bacteroides*) that can effectively metabolize and utilize the available nutrients. In addition, several members of genus *Bacteroides* play an important role in producing short chain fatty acids, which are known to be important for maintaining both intestinal homeostasis and bone health (Lucas et al., 2018; Hee and Wells, 2021). Of particular importance, there have been studies demonstrating that directly supplementing with *Bacteroides* species prevents bone loss in pre-clinical models as well as positive associations with bone health in animals (Villa et al., 2018; Yuan and Shen, 2021). In contrast, a recent meta-analysis revealed the genus *Bacteroides* is negatively associated with bone health in post-menopausal women (Huang et al., 2022). Of note is that the meta-analysis only included studies of post-menopausal women with sex-steroid deficiency-induced bone loss (primary osteoporosis), not those afflicted by GIO, which is mediated by a significantly different mechanism and as such, the responses in the gut microbiota may be different. In addition, the genus *Bacteroides* contains several unique species and strains, of which there are several important commensals and pathogens. As such, it is difficult to make definitive statements regarding a diverse group of bacteria and their health relevance. It is plausible that in the previous reports of negative associations between *Bacteroides* and bone health, specific bacteria that are more pathogenic are increased in response to estrogen deficiency. It is also plausible that in the case of our study, there is an increase in beneficial commensal *Bacteroides* species that associate positively with bone health. Unfortunately, without more accurate sequencing databases, we are unable to tease out these differences at this time. However, in this study, our results suggest a positive role for genus *Bacteroides* in the increased bone volume observed in response to DP treatment. Future studies will be necessary to determine if genus *Bacteroides* is important in mediating benefits to bone health, particularly in the context of GIO and DP supplementation.

Another genus of bacteria that was drastically altered and, in this case, reduced in response to DP supplementation was *Clostridium sensu stricto 1*, which is a group of anaerobic, fermenting bacteria containing several opportunistic human pathogens and some bacteria that have been utilized industrially for acetic acid and ethanol production (Li et al., 2023). In the random forest models, *Clostridium sensu stricto 1* was the third most important genus in both classifying samples into appropriate groups as well as in the regression model to predict BV/TV%. A recent meta-analysis analyzing gut 16S rRNA amplicon data from patients in China and South Korea revealed that *Clostridium sensu stricto 1* was significantly elevated in patients with osteoporosis (Akinsuyi and Roesch, 2023). To our knowledge, there have not been previous reports demonstrating the relation between *Clostridium sensu stricto 1* and GIO or DP supplementation. As such, our results are the first to demonstrate that DP treatment for 8 weeks in female mice reduces the relative abundance of *Clostridium sensu stricto 1* present within the fecal microbiome from approximately 10% to zero, and there is a strongly significant negative correlation between the relative abundance of *Clostridium sensu stricto 1* and distal femur trabecular BV/TV%. In addition, our results support what has previously been reported in the literature, with reductions in the relative abundance of *Clostridium sensu stricto 1* being associated with improvements in bone health (Akinsuyi and Roesch, 2023). Future studies will be necessary to better understand the role of *Clostridium sensu stricto 1* and the role that this group of bacteria plays in mediating bone health in not only this model of GIO, but bone health in general.

The mechanisms by which DP exert bone benefits are still unclear, although DP supplementation can lead to a reduction in bone turnover (Wallace, 2017). A major component from the prune that has been studied extensively regarding their mechanism are the polyphenols present within prunes. Polyphenols are compounds that naturally occur in several plant foods and contain one or more phenolic hydroxyl group (Tijjani et al., 2020). Over 8,000 unique polyphenols have been identified including flavonoids and phenolic acids, among several other classes. We analyzed approximately 30 of the most abundant flavonoid and phenolic acid compounds via mass spectrometry to develop a basic polyphenol and flavonoid fingerprint of the prune powder that we used (Table 2). Several phenolic compounds were elevated in the 25% prune diet, including chlorogenic acid, cinnamic acid, protocatechuic acid, quercetin-3-glucoside, and rutin. Each of the five compounds mentioned has been associated with prevention of bone loss in several models (Jang et al., 2018; Fayed et al., 2019; Mo et al., 2019; Baraka et al., 2022; Hong et al., 2022). Of note, chlorogenic acid (Mo et al., 2019) and rutin (Baraka et al., 2022) have been specifically implicated in preventing GIO in rats through alterations in the RANKL/OPG ratio and osteoclast number, respectively. In both cases, the responses favor reduced osteoclast activity, thus a reduction in early GC-induced bone loss (Chotiyarnwong and McCloskey, 2020). In this study, we did not directly test whether the polyphenols present in prune are mechanistically involved in mediating the protective effect of prunes on bone health in this model. However, given the previous body of literature relating specific polyphenols and bone health, and the significant amounts of polyphenols present in the 25% prune diet, it is plausible that the polyphenols present in prune may be mediating bone effects both in the prevention of GIO as well as the drastic improvement in healthy animals. Future studies will be necessary to investigate whether individual or groups of polyphenolic compounds from prune are sufficient to mediate the beneficial effects that are observed in this GIO model. Of note, Smith *et al.* recently noted that the polyphenol and carbohydrate fractions derived from prune crude extract each effectively prevented estrogen-deficiency osteoporosis in a mouse model, suggesting that both polyphenol and prebiotic activity are critical components of the DP effects on bone health (Smith et al., 2022). Given this, it is difficult to draw conclusions on whether one component or the other is exerting the main effect. It is currently unclear which component(s) of DP are involved in the bone health response and future studies will be necessary to identify the DP factors modulating bone health.

Taken together, this is the first study to demonstrate that DP supplementation effectively prevents bone loss and drastically alters gut microbiota composition in an established model of GIO in female mice. Future studies will further investigate the mechanism by which prunes exert a beneficial effect in this model and the role that the gut microbiota and its derived products play in mediating these effects. Our findings support the potential usefulness of DP to maximize bone density and use clinically as an adjunct to prolonged GC therapy to prevent its deleterious effects on bone health.

## Data Availability

The datasets present in this study can be found in online repositories. The names of the repository/repositories and accession numbers can be found below: https://qiita.ucsd.edu/public/?study_id=13961, Study ID 13961. https://qiita.ucsd.edu/analysis/description/46850/, Analysis ID 46850. EBI-ENA accession numbers: PRJEB72123 and ERP156903.
