# Supplementary figures and images for: Glucocorticoid-induced osteoporosis is prevented by dietary prune in female mice
Source: Front Cell Dev Biol. 2024 Feb 5;11:1324649. doi: 10.3389/fcell.2023.1324649 (PMC10875082; doi:10.3389/fcell.2023.1324649)

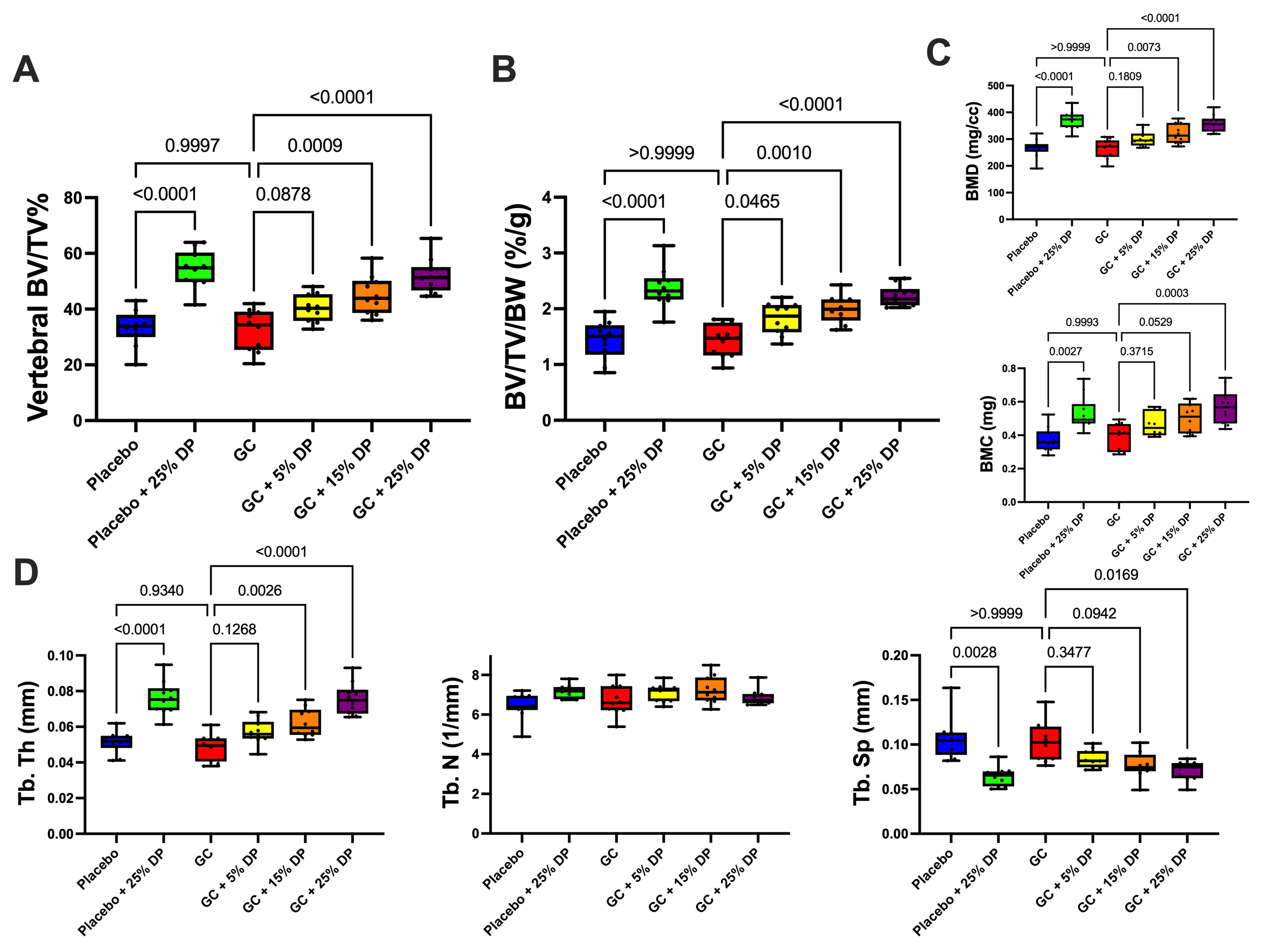

Supplement: Supplementary file 1 [file Image1.TIFF]
