# Supplementary material for: Glucocorticoid-induced osteoporosis is prevented by dietary prune in female mice
Source: Front Cell Dev Biol. 2024 Feb 5;11:1324649. doi: 10.3389/fcell.2023.1324649 (PMC10875082; doi:10.3389/fcell.2023.1324649)
Supplement: Supplementary file 2 [file DataSheet1.PDF]

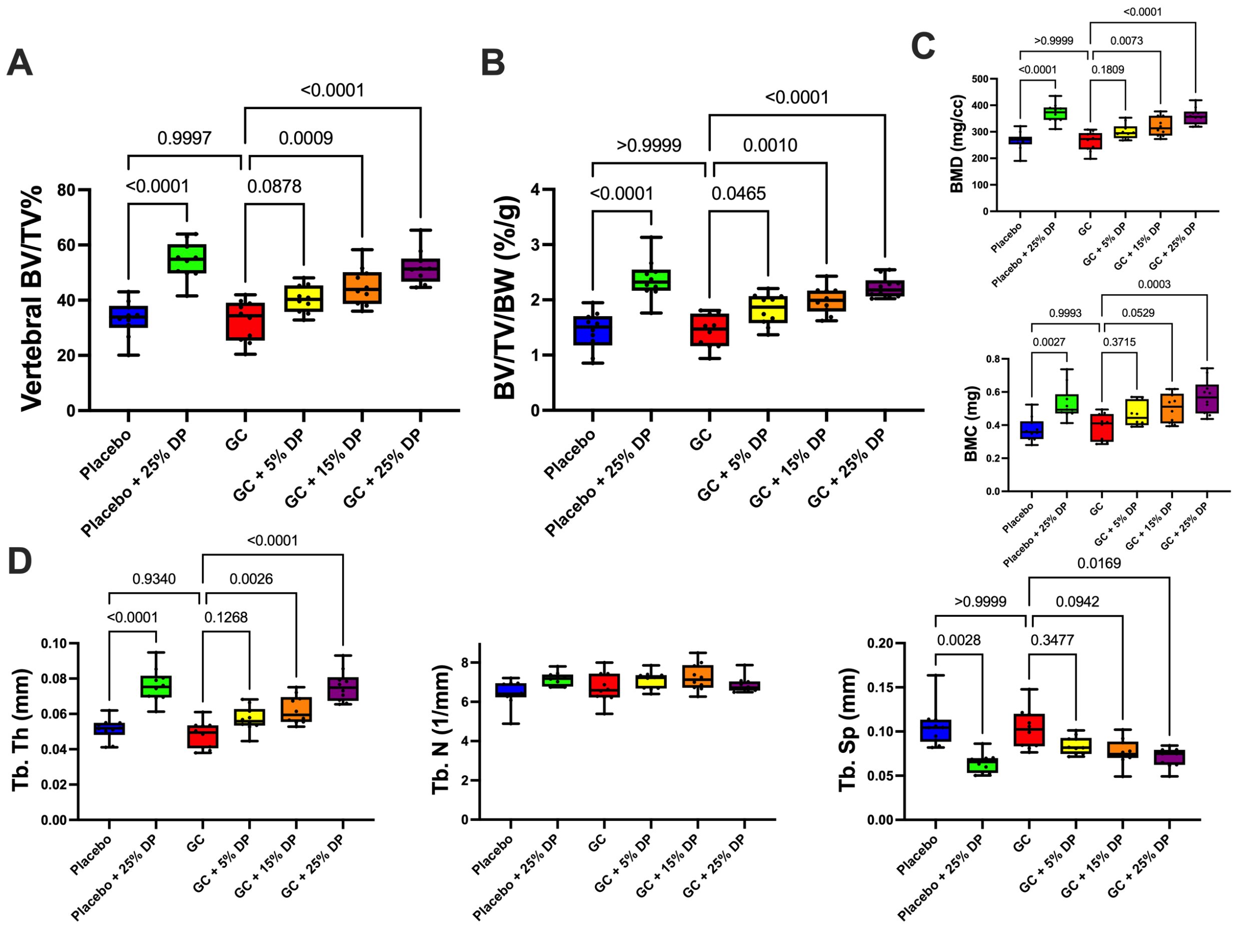

Figure S1. Dietary prune improves vertebral trabecular bone microarchitecture. 16-week-old female C57BL/6J mice were treated ± GC and ± prune supplemented diets (5, 15, and 25%) for eight weeks. μCT analysis of L4 vertebrae trabecular bone expressed as **(A)** bone volume fraction and **(B)** corrected for body weight. **(C)** Vertebral trabecular bone mineral density and bone mineral content. **(D)** Vertebral trabecular bone microarchitectural analyses. n = 10/group. Box and whisker plots represent the range of the data with lines at the median and quartiles. Statistical analyses performed via One-Way ANOVA. GC = glucocorticoid (Prednisolone), DP = dietary prune, BV/TV% = bone volume / total volume, BV/TV/BW = bone volume/total volume/body weight, BMD = bone mineral density, BMC = bone mineral content, Tb. Th. = trabecular thickness, Tb. N. = trabecular number, Tb. Sp. = trabecular spacing.
